# Role of mesenchymal cells in enhancing cosmetic outcomes for autologous augmented fat transfers for facial rejuvenation and reconstructive surgery

**DOI:** 10.3389/fmed.2024.1466939

**Published:** 2024-11-14

**Authors:** Soroush Ansari Lari, Maya Salem Zumot, Salsabiel Nemrish, Salim Fredericks

**Affiliations:** ^1^Royal College of Surgeons in Ireland- Medical University of Bahrain, Muharraq, Bahrain; ^2^Royal College of Surgeons in Ireland- Medical University of Bahrain, Muharraq, Bahrain; ^3^Royal College of Surgeons in Ireland- Medical University of Bahrain, Muharraq, Bahrain; ^4^Department of Biochemistry, Royal College of Surgeons in Ireland- Medical University of Bahrain, Muharraq, Bahrain

**Keywords:** autologous fat transfer, adipose-derived stem cells (ADSCs), volume retention, regenerative medicine, facial rejuvenation

## Abstract

In recent years, autologous fat transfer (AFT) has gained popularity for reconstructive and cosmetic procedures due to its minimally invasive nature and natural-looking results. However, limitations such as unpredictable fat resorption and safety concerns persist. To address these issues, researchers have explored incorporating adipose-derived stromal cells (ADSCs) into fat grafts. Enriching fat grafts with ADSCs, often through stromal vascular fraction (SVF), shows promise in regenerative medicine, though their effectiveness remains debated. Some studies suggest no significant difference in outcomes, while others indicate that ADSCs are more effective in larger-volume grafts. This implies that ADSC-enriched grafts might achieve similar results to traditional methods, with volume retention being a crucial success indicator. Given that these cosmetic procedures impact body image and self-confidence, innovative techniques like ADSC-enriched grafts are crucial for improving clinical outcomes. ADSCs are favoured for their abundance in adipose tissue and wound healing properties, which enhance cosmetic results. Patients receiving ADSC-enriched grafts show increased collagen, elastin, and CD31 levels, and better graft survival compared to those with traditional fat grafting, reducing the need for repeat procedures. Recent applications in patients with fibrotic facial deformities have demonstrated positive outcomes both cosmetically and psychologically. This mini-review evaluates the efficacy and benefits of ADSC-enriched AFT for facial rejuvenation and reconstruction, focusing on graft retention and overall procedural outcomes.

## 1 Introduction

Autologous fat transfer (AFT), or lipofilling, involves harvesting adipose tissue from the patient and reinjecting it beneath their skin to enhance or restore volume (1). It can be applied for both reconstructive and cosmetic purposes. The first documented adipose graft for cosmetic purposes was done by Czerny in 1895 to repair postmastectomy defects (2). Since then, it has been widely used for facial rejuvenation, breast contouring, and buttock augmentation (3). Autologous fat is considered the ideal filler for cosmetic procedures due to its biocompatibility, longevity, and soft and natural-looking results, which other fillers cannot replicate (4). The effectiveness of AFT can be improved by adding the stromal vascular fraction (SVF), which contains a heterogeneous mixture of cells such as adipose-derived stem cells, endothelial cells, pericytes, and immune cells. SVF enhances graft survival and vascularization, leading to better overall outcomes (5).

Extraction of SVF can be achieved mechanically, using enzyme-free methods by utilising devices such as the Rigenera® system. This method preserves the structural integrity of adipose tissue by maintaining the vascular stromal arrangement and perivascular cells. An experimental study conducted by De Francesco et al. revealed no significant differences in cell variability between enzymatic and mechanical SVF extraction, and in both methods, the cells maintained their capability of proliferating and maintaining stem cell characteristics (6).

**Figure 1:**
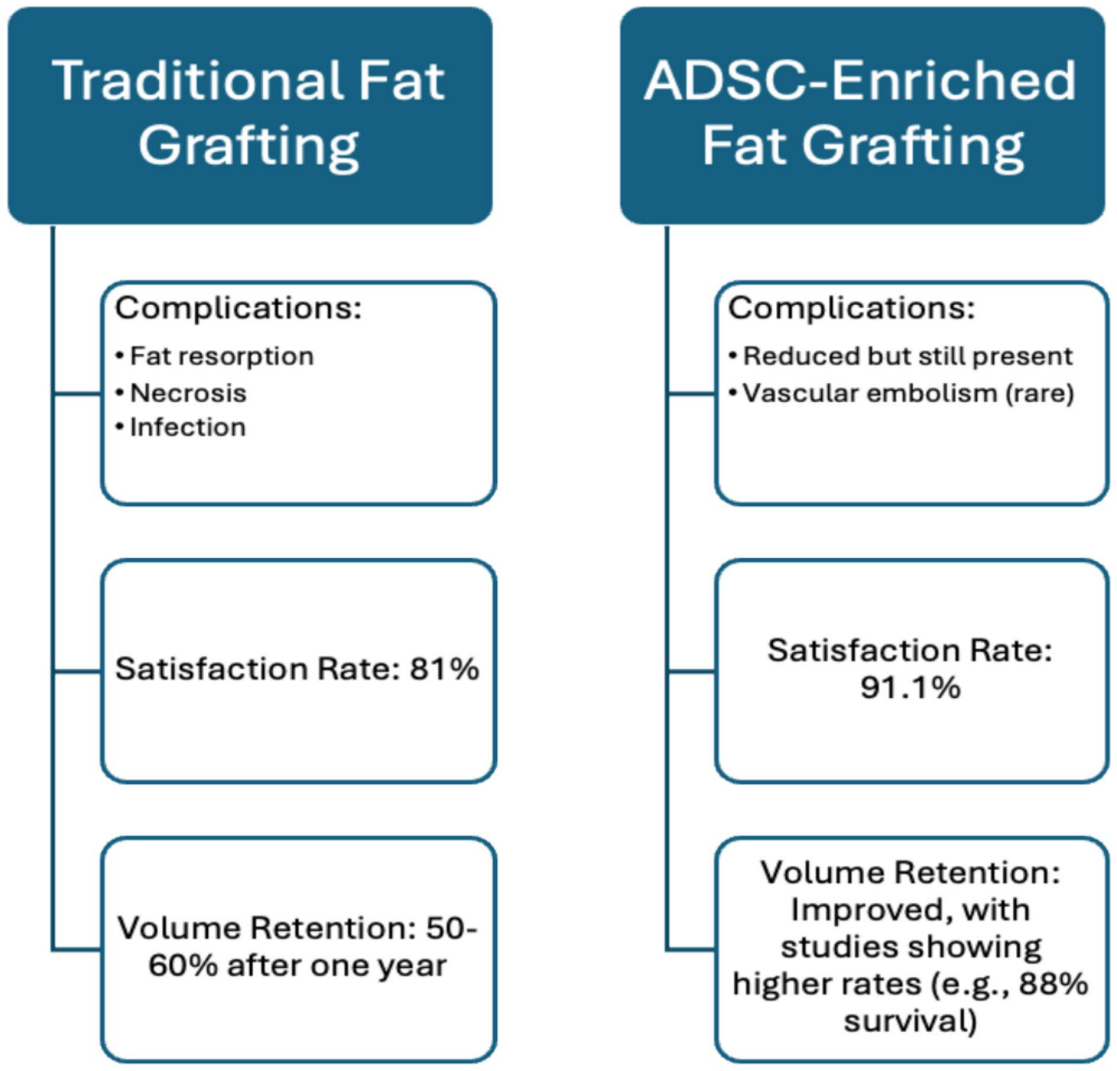
Comparison of traditional vs. ADSC-enriched fat grafting.

The mechanism of action of SVF (Figure 1) relies on the extraction of cells from adipose tissue with regenerative properties, including stem cells, immune cells, and endothelial cells (7). The extracted SVF is then paired with a specially prepared extracellular matrix (ECM) that allows the cells to thrive. The ECM is preconditioned with cobalt chloride to enhance the regenerative properties of SVF by promoting an increase in cell survival, adhesion, angiogenesis, and collagen production. The boost in angiogenesis is due to increased release of growth factors like vascular endothelial growth factor (VEGF), which is promoted by the hypoxic environment (8). The synergy between SVF and ECM has produced exceptional results. Sheng et al. demonstrated that SVF-gel formed an advanced ECM framework, significantly increasing the expression of TNF-*α* and VEGF. This enhancement promotes greater vascularization and adipocyte regeneration, both of which are critical factors for ensuring long-term volume retention (9). The fat cells, in turn, offer a long-lasting alternative to fillers used for cosmetic purposes, as the graft survives longer and enhances the volume and quality of the treated area (10).

Facial rejuvenation aims to address a myriad of changes that occur within the face as the human body ages. Wrinkles are unflattering folds and shadows that occur due to gravity; resuspension procedures such as face lifting can be employed to combat these gravity-related signs of ageing. Though resuspension procedures can provide a more youthful look by removing extra skin, decreased facial volume remains. This phenomenon occurs secondary to a combination of muscular atrophy, dermal thinning, and fat volume loss which progresses with ageing (11). Due to this, AFT and dermal fillers have risen in popularity as adjunctive treatments to combat the signs of ageing (4) (12).

Despite its advantages, AFT has limitations, notably, the unpredictability of volume maintenance due to varying rates of survival of the graft (13). Considering the application of fat grafting is to increase volume, techniques for maximising the survival and uptake of grafts are essential. Research on adipose-derived stem cells (ADSCs) shows promise in improving grafting outcomes, as their regenerative properties may enhance tissue integration and volume retention (14, 15).

Research on the application of ADSCs in fat grafting has shown optimistic outcomes. A 2022 study by Chen et al. found that ischemia in adipose tissue is a major barrier to graft survival. The research showed that adipose-derived stem cells (ADSCs) are valuable for promoting graft vascularization, which can significantly enhance graft survival by improving the supply of oxygen and nutrients (15). This vascularization process is largely mediated by the release of growth factors, such as VEGF and FGF (fibroblast growth factor), which are crucial for angiogenesis and tissue regeneration. These growth factors not only facilitate the formation of new blood vessels but also play a role in cell rejuvenation, contributing to the longevity and effectiveness of the graft (16–18). However, questions remain regarding the overall efficacy and optimal methods of application. Past studies on ADSCs for cell-assisted lipotransfer (CAL) have been scrutinised mostly for lacking control groups for objective comparison, relying instead on subjective assessments through image analysis and clinical examination (19).

In this mini-review, we aim to reach a consensus on the efficacy and benefits of the addition of adipose-derived stromal cells to the preparation of autologous fat grafts for facial rejuvenation and reconstruction.

## 2 Adipose tissue and autologous fat grafting

Adipose tissue harvesting and autologous grafting have become more popular in recent decades due to their low immunogenicity, cost-effectiveness, and easy accessibility (20). It can also provide autologous reconstruction using minimally invasive techniques (21).

AFT has high satisfaction rates—91.1% among patients and 88.6% among surgeons, according to Krastey et al.’s systematic review. However, questions remain about its long-term reliability, especially regarding volume retention. Krastey et al. found that, on average, 1.5 fat transfers were needed to achieve desired results, with only 50–60% of the injected volume retained after 1 year (21). These results demonstrate AFT’s effectiveness and reliability, as the review included 51 studies and 1,533 patients. These findings highlight the need to use objective measures, such as MRI or 3D surface imaging to measure the percentage of fat retained over time (ex. at 3, 6, and 12 months) (22). To achieve more standardised data on patient satisfaction, tools like the FACE-Q questionnaire can be employed. This questionnaire quantifies patient satisfaction across key domains, including aesthetics, functional outcomes, and overall quality of life, allowing for consistent assessment over time (23). The inclusion of four randomised controlled trials (RCTs), the gold standard for establishing causality, strengthens Krastey et al.’s conclusions. However, the generalizability may be limited due to the specific patient populations studied, including a predominance of those with HIV-associated lipodystrophy and less common conditions like Parry-Romberg syndrome. Additionally, reliance on subjective data collection, such as patient questionnaires, introduces variability that could affect the results’ reliability. Future studies should involve more diverse patient populations with common facial deformities, like scars and burns, and use advanced imaging techniques to measure fat retention and graft survival objectively over time. This approach would enable a more accurate assessment of AFT’s efficacy and safety, improving its clinical application.

Another systematic review by Groen et al. also found that fat grafting has great potential, with a low complication rate of 6% and a high patient satisfaction rate of 81%, highlighting its overall efficacy (24). The results of this systematic review are reliable because they accounted for bias and heterogeneity among the included studies. The review included 18 clinical articles and 3,073 patients, providing a large cohort that enhances the accuracy of the results. However, limitations of this review include reliance on low-level evidence due to the lack of published research on the topic. Additionally, the studies used different fat cell harvesting techniques, which could contribute to variations in resorption rates.

As noted, AFT is generally effective and low-risk, but if complications occur, they can range from mild skin irregularities to severe fat necrosis (25). A systematic review by Brucato et al. which looked at the complications associated with facial AFT found that most severe complications, accounting for 53%, were related to injections in the temporal area (26). Reported complications from this review include lipogranuloma, mycobacterial abscess, hemiplegia, vision loss, and vascular embolism, highlighting the severity of complications that can arise from fat grafting. The review’s results are reliable, as it examined 22 different articles involving 1,205 patients, providing an adequate cohort for the meta-analysis. The review found that, of the 1,205 patients, only 38 reported complications, totalling 58 complications. These figures may reflect either underreporting of complications or an accurate complication rate. This meta-analysis could not determine the cause due to limitations in the included studies, including low evidence scores and heterogeneity.

## 3 Comparing complications between traditional, and stromal-cell-enriched fat grafting techniques

A review by Schultz et al. discusses complications associated with traditional fat grafting, including fat resorption, necrosis leading to oil cysts or calcifications, infection, and fat embolisms which is a rare but severe complication (27). The paper provides a comprehensive review through long-term follow-ups, offering insight into the procedure’s longevity and effectiveness. However, technique variability limits definitive conclusions.

In a retrospective review, Fang et al. assessed the safety of traditional fat grafting techniques, finding a 27.8% complication rate, with 10.9% classified as major and 16.7% as minor. Major complications included hematoma/seroma (2.5%), dermatitis/cellulitis (3.3%), and infection (1.8%). Minor complications included asymmetry (14.4%), altered sensation, and pain. No severe complications like blindness, stroke, or intravascular issues were reported (28). The study’s large sample size (n = 396) and its application across multiple grafting sites for both cosmetic and reconstructive purposes enhance its value. However, its retrospective design limits the ability to establish causation and lacks long-term data.

An experimental study by Chen et al. combined *in vivo* and *in vitro* approaches, examining AFT with ADSC-enriched exosomes. Although ADSC-derived exosomes offer promising benefits for graft survival and promote adipogenic differentiation, common complications persist. The study observed improved graft retention and vascularization with ADSC-derived exosomes compared to human foreskin fibroblast exosomes, but both groups experienced a gradual decrease in graft weight and volume over time. Complications such as cyst formation, calcification, nodules, fat necrosis, and fibrosis were noted (29). While ADSC-derived exosomes showed superior graft retention, the complications highlight ongoing concerns about graft stability (30). The study’s innovative approach is promising but limited by its small sample size, short-term focus, and reliance on a murine model.

A review by Crowley et al. focused on stromal cell-enriched fat grafting techniques for facial rejuvenation, examining the use of ADSCs, stromal vascular fraction (SVF), and nanofat. Key complications include variability in fat graft survival rates, which range from 25 to 70%, influenced by viable adipocytes and grafting conditions. Challenges in integrating stem cells, including dose and timing effects on graft outcomes, were also highlighted (31). The review’s strengths include its exploration of innovative techniques and a comprehensive overview of emerging regenerative therapies. However, it notes limitations such as a lack of extensive clinical evidence and regulatory approval for many methods discussed. The focus on less established techniques may leave well-established methods underrepresented.

Exploring the complications of traditional and stromal cell enriched techniques underscores that both have significant complications. The superiority of stromal cell enriched techniques is explored to assess how much they reduce complications rather than eliminate them entirely, as they are inevitable due to the procedure’s nature. More emphasis is needed on exploring each technique individually and quantifying the complications.

## 4 Adipose-derived stromal cells and autologous fat grafting

Autologous fat aspirates undergo several steps to become enriched with ADSCs. Fat is harvested from the donor area using a low vacuum pressure syringe and then washed in a closed system to avoid contact with air. Stromal cells are isolated via enzymatic digestion of the fat, or enzymatic-free techniques; this produces a high density SVF (19). The fat sample to be grafted is then enriched with SVF to then be used for CAL (32, 33).

Both human and animal trials have targeted the efficacy of CAL in fat graft survival. The focus has been on reconstructive purposes like treating lipodystrophic diseases, like hemifacial atrophy, and craniofacial microsomia, but also for cosmetic procedures like scar revision and facial contouring (34).

An animal model utilising fluorescence-stained SVF to monitor the survival of a stromal cell enriched graft was used to investigate the fate of implanted SVF. Over 56 days, the most significant decrease in fluorescence intensity occurred in the first 14 days; by the end of the monitoring period, the remaining signal was 17.3% of the initial level (35). The results outline that implanted SVF can survive the ischemic environment of fat grafts and aid in adipogenesis and angiogenesis. However, rodent models differ significantly from humans in physiology, size, and wound healing processes, reducing the external validity of this animal research (36).

Human studies comparing CAL to conventional methods of adipose tissue grafting have shown results. Regenerative properties of ADSCs were first demonstrated in a clinical study on 19 patients with radiation-induced soft-tissue damage. ADSC-enriched lipoaspirate transplants significantly improved symptoms, with 4 of 11 patients with irreversible functional damage becoming symptom-free and all but one experiencing some degree of improvement (37). The study’s strengths include long-term follow-up over 31 months and a prospective cohort design that allows for risk factor identification, essential for exploring new technologies. However, the small sample size and lack of a control group limit the generalizability of the findings and make it difficult to attribute improvements solely to the treatment.

A systematic review and meta-analysis by Li et al. compared conventional lipotransfer versus CAL for breast augmentation, highlighting CAL’s superiority in certain aspects. Pooled effect estimates indicated that CAL was superior in fat survival (SMD = 1.79, 95% CI = 0.28, 3.31; *p* = 0.02), achieving statistical significance. However, no significant differences were found in complication rates between CAL and conventional methods, and subgroup analysis showed no significant differences in fat survival between SVF-enhanced and conventional grafts (38). This review benefits from robust quantitative measures in assessing CAL’s effectiveness, but the relatively small sample size (*n* = 353) limits generalizability, and significant heterogeneity among the studies affects the findings’ reliability. Although not focused on facial rejuvenation, its emphasis on graft survival is valuable for comparing the two techniques.

A study comparing conventional lipoinjections and CAL for facial lipoatrophy found that the CAL group showed greater clinical improvement. Four blinded certified plastic surgeons assessed pre- and post-operative photographs of patients, noting ≥60% volumetric improvement in the CAL group, while the non-CAL group showed 40–60% improvement; one patient in the non-CAL group experienced adipose necrosis (39). Using a prospective comparative study allows for comparisons between CAL and conventional lipoinjection techniques, providing insights into the safety and efficacy of the new technique. Additionally, the clinical improvement scoring system adds robustness to the results. However, this study did not achieve statistical significance (*p* = 0.11), weakening the conclusion regarding the superiority of CAL.

A literature review conducted by Moustaki et al. concluded that preliminary studies show promising evidence that ADSCs may improve volume-restoring and retention capabilities of transplanted fat (19). The use of CAL was shown to be superior to conventional methods of fat grafting in the reviewed literature; for instance, Tanikawa et al. reported a fat volume survival rate of 88% with CAL compared to 54% without CAL at 6 months post-procedure (40). Additionally, Kølle et al. conducted a randomised controlled trial assessing CAL with *ex vivo* expanded ADSCs, achieving a residual volume of 81%, in contrast to 16% in the non-CAL group. These included studies demonstrate the ability of adipose-derived stem cells to promote neovascularization and tissue regeneration, which is crucial for achieving natural and lasting facial volume improvements (41). The literature review benefits from including randomised controlled trials, providing robust evidence for CAL’s superiority over traditional lipoinjections while minimising bias and ensuring high methodological quality. The clinical relevance of this review is paramount as it focuses on patient satisfaction and aesthetic outcomes, which is often a decisive factor when considering elective cosmetic procedures. However, the studies cited often feature small sample sizes, limiting statistical power and generalizability. Moreover, because of the novel application of ADSCs in enriching fat grafts, there is variability in techniques used across studies, introducing variability in outcomes and complicating direct comparisons.

**Figure 2:**
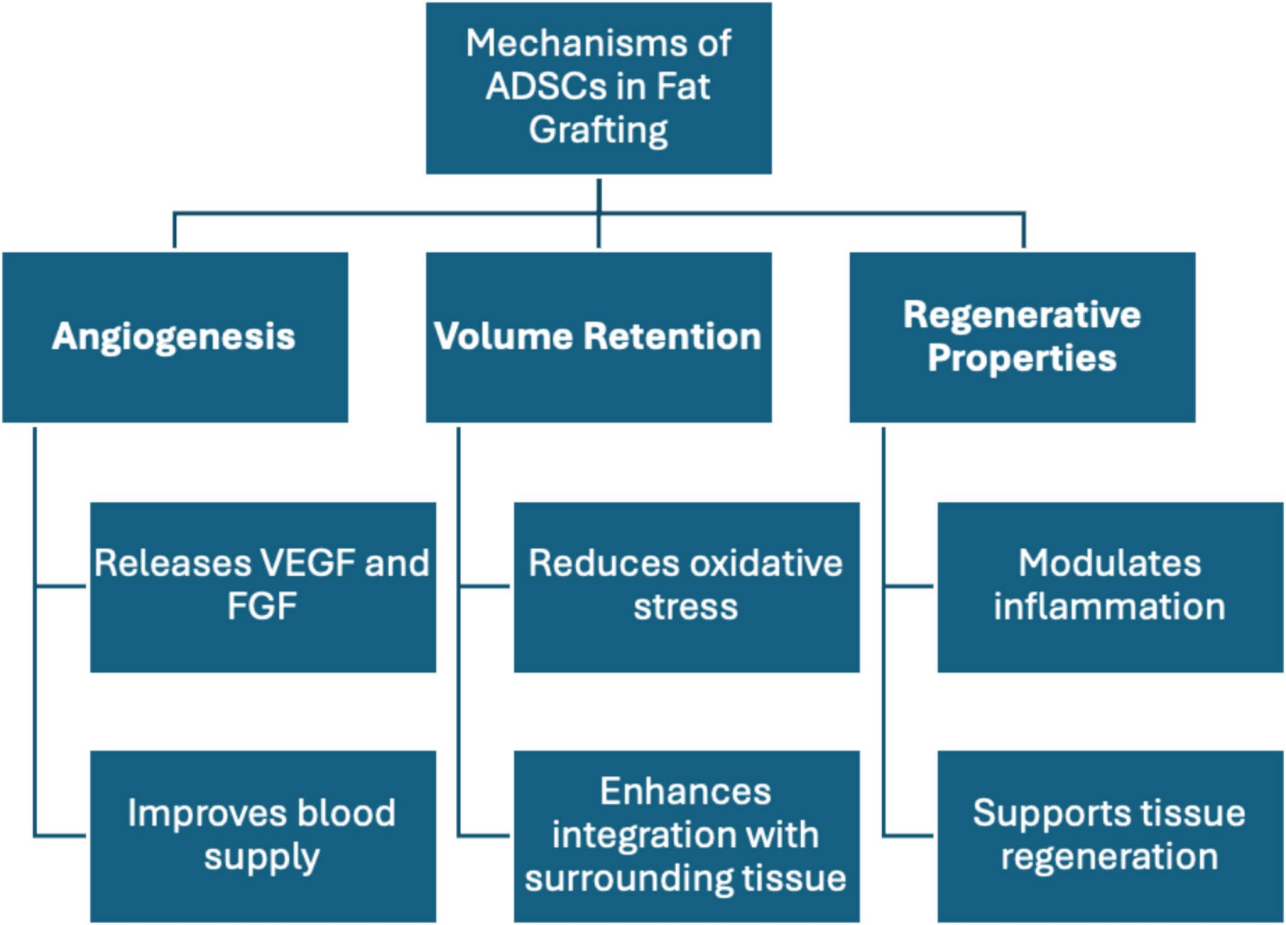
Mechanisms of adipose-derived stromal cells (ADSCs) in fat grafting.

A systematic review by Qin et al. explored the role of ADSCs in regenerative medicine, focusing on the physiological effects of CAL. A study by Chen et al. demonstrated that ADSCs improve fat graft survival by modulating oxidative and inflammatory responses through the TLR4 and Nrf2 pathways (42). ADSCs enhance fat graft survival by reducing oxidative stress, promoting angiogenesis through the Nrf2 pathway, and modulating inflammation via the TLR4 pathway, creating a supportive environment for graft viability and integration (43) (Figure 2). Another murine study included in the review, conducted by Yu et al., focused on transfecting ADSCs with modified mRNA encoding VEGF, enhancing their proangiogenic ability and improving graft survival (44). ADSCs contribute to graft survival primarily through paracrine effects, such as secreting VEGF, and by differentiating into endothelial cells that support angiogenesis, mitigating and reversing necrosis of the grafted fat. VEGF is key for neovascularization, promoting endothelial cell proliferation, and inhibiting apoptosis. Additionally, VEGF works synergistically with factors like FGF-2 and HIF-1α, which promote fat survival and integration, especially in hypoxic or inflammatory states common after grafting (18). A study by Borrelli et al. found that the CD34+ and CD146+ subpopulation of ADSCs exhibited enhanced graft survival due to higher levels of proangiogenic factors like VEGF, fibroblast growth factors, and angiopoietin-1. This suggests that using these specific ADSC subgroups could improve graft survival (45) (Figure 2). Although the review covered multiple applications of ADSCs in regenerative medicine, it highlighted the need for more guidelines to ensure consistent ADSC isolation, essential for comparing studies. The optimistic tone of the review may reflect publication bias, as unsuccessful or opposing insights were lacking. The review’s deep exploration of pathways aiding graft survival was notable, though the reliance on murine studies limits generalizability. Moreover, the human trials in the review would benefit from larger sample sizes to increase statistical power and enhance confidence in the conclusions. Overall, the implications of these findings extend beyond individual studies, underscoring the potential for AFT to enhance both reconstructive and cosmetic surgical outcomes.

## 5 Discussion

AFT has gained significant traction for a range of applications, particularly in facial rejuvenation. It offers a minimally invasive option with a low risk of immune reactions, yielding natural results. Despite high patient satisfaction and generally positive outcomes, AFT has limitations and potential complications that warrant consideration.

A major concern with AFT is the unpredictability of volume resorption over time, making it less suitable for patients unable to commit to follow-up procedures. While complication rates are generally low, issues such as necrosis and asymmetry can arise. Additionally, various procedural techniques and adjuvant therapies, including botulinum toxin and fillers, may optimize results but do not eliminate complication risk.

To address these challenges, ADSCs have been investigated. Autologous fat aspirates undergo several steps to enrich ADSCs. Fat is harvested using a low vacuum pressure syringe, washed in a closed system to prevent air exposure, and then subjected to enzymatic digestion and centrifugation to isolate a high-density SVF. Research demonstrates that CAL can enhance graft survival, improving volume retention compared to traditional methods. ADSCs may improve fat graft survival by modulating oxidative and inflammatory responses, enhancing angiogenesis, and creating a supportive environment for graft viability.

While murine models suggest positive outcomes, concerns exist regarding their external validity due to differences in human physiology and healing processes. Human trials have shown promising results, with substantial improvements in graft survival and aesthetic outcomes. However, many studies are limited by small sample sizes, lack of control groups, and variability in fat grafting techniques, complicating comparisons and undermining generalizability. Furthermore, reliance on subjective questionnaires introduces potential bias, highlighting the need for systematic reviews in the context of CAL.

Larger-scale multicentre randomized controlled trials (RCTs) are necessary to evaluate the efficacy and safety of CAL, addressing concerns such as the theoretical risks of tumorigenicity associated with ADSC properties. Future studies should implement rigorous statistical methods and report confidence intervals and *p*-values clearly to strengthen conclusions about CAL’s efficacy. Multivariate analyses can help control for confounding variables and enhance the reliability of outcomes.

In conclusion, while ADSCs show promising potential in AFT for facial rejuvenation and reconstruction, significant gaps remain in the research. Uncertainties regarding long-term effectiveness, safety, and standardisation of techniques necessitate rigorous investigation. Well-designed RCTs with larger sample sizes and standardised methods for ADSC processing are essential. Incorporating objective measurements like volume retention and fat resorption rates, along with advanced imaging techniques such as 3D volumetric analysis and patient-reported outcome tools like the FACE-Q questionnaire, will refine evaluations and guide clinical practice. Addressing these challenges will enhance the safety and efficacy of AFT, ultimately improving patient outcomes in both cosmetic and reconstructive applications.
