# Supplementary material for: Role of mesenchymal cells in enhancing cosmetic outcomes for autologous augmented fat transfers for facial rejuvenation and reconstructive surgery
Source: Front Med (Lausanne). 2024 Nov 14;11:1466939. doi: 10.3389/fmed.2024.1466939 (PMC11602321; doi:10.3389/fmed.2024.1466939)
Supplement: Supplementary file 1 [file Data_Sheet_1.PDF]

ChatGPT 4.0 was used to identify complex sentences and to reword certain things in a more straightforward manner; prompts used are highlighted below, and the outputs:

reword this: SVF-gel provided a better ECM framework and enhanced the expression of VEGF and TNF-α in the grafted fat, leading to increased vascularization and adipocyte regeneration, both of which are of great importance in long-term volume retention of the fat grafts.

SVF-gel created a superior ECM framework and boosted the expression of VEGF and TNF-α in the grafted fat, resulting in enhanced vascularization and adipocyte regeneration. These factors are crucial for the long-term retention of volume in fat grafts.

Identify the sentences which are complex and difficult to read: 1. Introduction

Autologous fat grafting, or lipofilling, involves harvesting adipose tissue from the patient and reinjecting it beneath their skin to enhance or restore volume (1). It can be applied for both reconstructive and cosmetic purposes. The first documented true adipose graft done for cosmetic purposes was done in 1893 to fill atrophic scars (2). Since then, it has been widely used for facial rejuvenation, breast contouring, and buttock augmentation (3). Autologous fat is considered the ideal filler for cosmetic procedures due to its biocompatibility, longevity, and soft and natural-looking results, which other fillers can't replicate (4).

Facial rejuvenation aims to address the myriad of changes that occur within the face as the human body ages. Wrinkles are unflattering folds and shadows that occur due to gravity; resuspension procedures such as face lifting can be employed to combat these gravity-related signs of ageing. Though resuspension procedures can provide a more youthful look by removing extra skin, decreased facial volume remains. This phenomenon occurs secondary to a combination of muscular atrophy, dermal thinning, and fat volume loss which progresses with ageing (5). Due to this, autologous fat grafting and dermal fillers have risen in popularity as adjunctive treatments to co[illegible]t the signs of ageing (4).

Despite its advantages, autologous fat grafting has limitations, notably, the unpredictability of volume maintenance due to varying rates of survival of the graft (6). Considering the application of fat grafting is to increase volume, techniques for maximising the survival and uptake of grafts are essential. Research on adipose-derived stem cells (ADSCs) shows promise in improving grafting outcomes, as their regenerative properties may enhance tissue integration and volume retention (7)(8).

Research on the application of ADSCs in fat grafting has shown optimistic outcomes. A 2022 study by Chen et al. identified adipose tissue ischemia as the main obstacle to graft survival and highlighted the potential of ADSCs in promoting vascularization, which may improve graft survival (9). However, questions remain regarding the overall efficacy and optimal methods of application. Past studies on ADSCs for cell-assisted lipotransfer (CAL) have been scrutinized mostly for lacking control groups for objective comparison, relying instead on subjective assessments through image analysis and clinical examination (10).

In this mini-review, we aim to reach a consensus on the efficacy and benefits of the addition of adipose-derived stem cells to the preparation of autologous fat grafts for facial rejuvenation and reconstruction.

Here are the sentences that are relatively complex and may be difficult to read:

1. "Autologous fat is considered the ideal filler for cosmetic procedures due to its biocompatibility, longevity, and soft and natural-looking results, which other fillers can't replicate."
   - This sentence contains multiple clauses and a list of characteristics that might be simplified for clarity.
2. "Wrinkles are unflattering folds and shadows that occur due to gravity; resuspension procedures such as face lifting can be employed to combat these gravity-related signs of ageing."
   - The sentence could be broken into smaller, more straightforward statements to improve readability.
3. "This phenomenon occurs secondary to a combination of muscular atrophy, dermal thinning, and fat volume loss which progresses with ageing."
   - The phrase "occurs secondary to a combination of" can be made simpler for easier understanding.
4. "Despite its advantages, autologous fat grafting has limitations, notably, the unpredictability of volume maintenance due to varying rates of survival of the graft."
   - This sentence includes technical language and multiple concepts, making it harder to digest quickly.
5. "Considering the application of fat grafting is to increase volume, techniques for maximising the survival and uptake of grafts are essential."
   - The sentence structure can be simplified by splitting the ideas into separate sentences.
6. "Research on adipose-derived stem cells (ADSCs) shows promise in improving grafting outcomes, as their regenerative properties may enhance tissue integration and volume retention."
   - The inclusion of multiple scientific terms and concepts in one sentence makes it denser and more difficult to read.
7. "However, questions remain regarding the overall efficacy and optimal methods of application."
   - This is a shorter sentence but is somewhat vague and could be clearer with more specific language.
